# Factors influencing trainee doctor emigration in a high income country: a mixed methods study

**DOI:** 10.1186/s12960-017-0239-7

**Published:** 2017-09-25

**Authors:** Nicholas Clarke, Sophie Crowe, Niamh Humphries, Ronan Conroy, Simon O’Hare, Paul Kavanagh, Ruairi Brugha

**Affiliations:** 1 Department of Epidemiology and Public Health Medicine, RCSI, Beaux Lane House, Mercer Street, Dublin 2, Ireland; 2 Royal College of Physicians of Ireland, Dublin, Ireland; 3 Medical Council of Ireland, Dublin, Ireland; 4 Health Service Executive, Dublin, Ireland

**Keywords:** Doctor emigration, Postgraduate, Training, Mixed-methods

## Abstract

**Background:**

The Global Code of Practice on the International Recruitment of Health Personnel focuses particularly on migration of doctors from low- and middle-income countries. Less is understood about migration from high-income countries. Recession has impacted several European countries in recent years, and in some cases emigration has reached unprecedented levels. This study measures and explores the predictors of trainee doctor emigration from Ireland.

**Methods:**

Using a partially mixed sequential dominant (quantitative) study design, a nationally representative sample of 893 trainee doctors was invited to complete an online survey. Of the 523 who responded (58.6% response rate), 423 were still in Ireland and responded to questions on factors influencing intention to practice medicine abroad and are the subjects of this study. Explanatory factors for intention to practice medicine in Ireland in the foreseeable future, the primary outcome, included demographic variables and experiences of working within the Irish health system. Associations were examined using univariable and multivariable logistic regression to estimate odds ratios for factors influencing the primary outcome. Qualitative interviews were conducted with 50 trainee doctors and analysed thematically, exploring issues associated with intention to practice medicine abroad.

**Results:**

There were high levels of dissatisfaction among trainee doctors around working conditions, training and career progression opportunities in Ireland. However, most factors did not discriminate between intention to leave or stay. Factors that did predict intention to leave included dissatisfaction with one’s work-life balance (odds ratio (OR) 2.51; 95% confidence interval (CI) 1.53–4.10; *P* < 0.001); feeling that the quality of training in Ireland was poor (OR 1.82; 95% CI 1.09–3.05; *P* = 0.002) and leaving for family or personal reasons (OR 1.85; 95% CI 1.08–3.17; *P* = 0.027). Qualitative findings illustrated the stress of doing postgraduate training with inadequate supervision, lack of ring-fenced training time and pressures on personal and family life.

**Conclusions:**

Large-scale dissatisfaction with working, training and career opportunities point to systemic factors that need to be addressed by health workforce planners if Ireland is to retain and benefit from a motivated medical workforce, given trainees’ perceptions that there are better opportunities abroad.

**Electronic supplementary material:**

The online version of this article (10.1186/s12960-017-0239-7) contains supplementary material, which is available to authorized users.

## Background

The WHO Global Code of Practice on the International Recruitment of Health Personnel was primarily a response to the emigration of doctors, nurses and other health professionals from low- and middle-income countries (LMICs) to high-income countries [1]. While now recognised as a global crisis [2], the migration of doctors is not a new phenomenon nor has its impact been restricted to LMICs. Large-scale migration of doctors from the United Kingdom (UK) to the United States of America (USA) was reported in the 1950s and 1960s, as UK doctors left in search of better opportunities, career progression and specialist training [3–5]. A similar phenomenon was reported in Ireland during the same period [6]; currently, specialist trainees (medical graduates undertaking postgraduate training) spend time abroad to gain subspecialty exposure that Ireland, with its small population, does not provide [7]. The outward migration of doctors to seek specialist training in international centres of specialism is seen as beneficial [7, 8], if the migration is “circular” and graduates return to the source country with valuable experience [1].

However, Ireland [9] and other high-income countries significantly impacted by the 2008 global economic crisis have seen high levels of emigration by doctors in recent years [10–13]. Research from Austria [14], Ireland [13] and Iceland [12] cite low salaries, long working hours and poor career progression as reasons for high levels of doctor emigration. A 2015 qualitative study reported that the main reasons for migration of doctors was the quest for better working conditions, career progression and better practice environments [13]. High levels of outward migration of medical graduates from the UK [15] and Ireland [13] result in service gaps and lead to high levels of inward migration [16]. While Ireland has doubled its domestic production of medical graduates to achieve self-sufficiency [17, 18] and produces 21.9 medical graduates per 100 000 inhabitants, the highest of all OECD countries in 2014 [19], it remains heavily reliant on foreign trained doctors, ranging from 31 to 36% of those registered on its Medical Council during 2010–2015 [20–22]. This level of reliance on foreign trained doctors is widely recognised as undesirable, unsustainable and at odds with Ireland’s commitment to self-sufficiency and to the WHO Global Code [1, 17, 23].

The emigration of doctors from high-income countries has been less studied and is less well understood than emigration from LMICs. Yet, it is no less important, not only because of its economic costs and impact on the source country but also because such high-income source countries often become destination countries for doctors from LMICs [21]. Ireland’s health system underwent major budget cuts following the economic recession in 2009, with the national Health Service Executive budget reduced by 22% and public health sector staffing by 10% (12 200 whole time equivalents) [9]; economic growth was re-established by 2014. Research has reported a broad range of systemic problems that explain the emigration of doctors from Ireland [13, 18, 23, 24]; a series of measures were recommended and adopted in 2014 by Ireland’s Department of Health to tackle these systemic problems. However, reports monitoring the implementation of findings show that trainees are yet to see improvements on the ground [25, 26].

Understanding the predictors of doctor emigration from high-income countries can inform measures to improve doctor retention, thereby also reducing recruitment from poorer source countries. This study builds on an earlier study of doctors who had left Ireland [13], which reported that an increasing proportion were choosing not to return [18, 27]. By interviewing and surveying a representative sample of trainee doctors still in training posts in Ireland [27], the paper aims to provide medical workforce decision-makers with evidence of the early impact of the 2014 measures to encourage their retention (e.g. protected training time, family friendly arrangements and mentoring among others), and analyse and report the phenomenon of medical migration between high-income countries to a wider audience. A mixed methods approach comprises quantitative estimates of migration intentionality and associations with factors that might influence emigration, from a self-administered survey, together with data from in-depth interviews to explore those factors. The use of a mixed methods approach increases the breadth and depth of understanding of either approach alone [28].

## Methods

This study employed a partially mixed sequential dominant (quantitative) study design [29]. Qualitative face-to-face and telephone interviews were conducted with doctors who were currently enrolled or had recently completed postgraduate training in Ireland. Interviews explored issues associated with intention to practice medicine abroad. A quantitative online survey of a nationally representative sample of doctors investigated the factors associated with migration intentions. Both studies were informed by previous research by the authors on health professional emigration [13, 18]. The study was approved by the host institution’s Research Ethics Committee.

### Sample

During 2014, the Medical Council of Ireland conducted an annual online survey of all circa 3000 trainees, which excluded doctors not within structured post-graduate training programmes [30]. The “Your Training Counts” (YTC) survey invited respondents to consent to an independent follow-up study involving face to face interviews and a survey by an independent research team. The YTC response rate was 53% (*n* = 1636 responses), of whom 56% (*n* = 912) agreed to be contacted for the Doctor Emigration Project. Of these, 893 (27 did not provide a valid email address, and eight were traced through a valid phone number) formed our final sample.

### Qualitative and quantitative phases

For the qualitative phase, a purposive sample was drawn from the list of consenting trainees, which was stratified by sex and location (working in Ireland or abroad) but which also aimed to achieve diversity in speciality and stage of training. Email invitations were sent to a sample of 342 doctors, and in-depth semi-structured interviews were carried out, in person or by telephone, with those who agreed to participate. For the quantitative phase, an online survey was developed, followed by consultation with stakeholders and piloting of the instrument with trainees. For the quantitative phase, a link to the survey and participant information was sent to the 893 trainee doctors who had consented to follow-up, with two reminder emails.

**Fig. 1:**
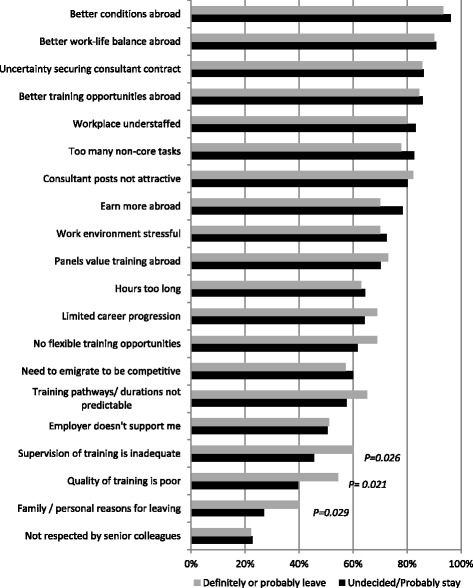
Percentage of participants in agreement with statements that they consider would influence their decision to practice medicine abroad. Those who stated they were “definitely remaining” were not required to respond to these statements. See Additional file 1 for full description of statements

The key outcome variable, intention to practice medicine in Ireland for the foreseeable future, was measured using a Likert scale (five response categories: No definitely not, No probably not, Undecided, Yes Probably, Yes definitely). Further questions were asked about destination country and likelihood of return. The survey included 20 Likert scale statements covering factors influencing trainees’ decisions to practice medicine abroad, which were developed from a review of the literature and previous work by the research team [13, 18]. These covered working conditions, training, career progression and personal factors (see Fig. 1 and Additional file 1: Table S1) with responses ranging from strongly agree to strongly disagree. Doctors who had left Ireland between 2014 and 2016 were not invited to respond to these intention-to-practice-abroad statements and therefore are not included in this analysis.

### Data collection and analysis

In-depth interviews took place during May–June 2015. Following informed consent, interviews lasted on average 60 min and were audio recorded and transcribed verbatim. All transcripts were entered into MAXQDA software, analysed by two authors (NH & SC) using thematic analysis [31] and then coded independently by both authors. The quantitative online survey was run during April and May 2016.

Quantitative data were analysed in Stata 14. We measured associations of the demographic and medical practice variables with the primary outcome variable, intentionality to practice medicine in Ireland using chi-square tests. Univariable and multivariable logistic regression analyses were used to estimate odds ratios for factors influencing the primary outcome (migration intentionality). Intentionality was recoded as a binary outcome comparing “Stayers” (definitely or probably intending to practice medicine in Ireland for the foreseeable future) and “Leavers” (definitely not or probably not intending to practice medicine in Ireland for the foreseeable future).

A secondary analysis compared “Leavers” to those who were “Undecided”, which included those who “Probably intend to stay in Ireland for the foreseeable future” and those who reported being “Undecided”. In this secondary analysis, we included Likert scale questions on factors influencing one’s decision to practice medicine abroad—those who definitely intended to stay in Ireland were not asked to answer these questions. Likert scale questions were treated as categorical and were further recoded (from four categories [Agree strongly, Agree, disagree and Disagree strongly] to two categories [Agree or Disagree]), making these binary variables.

## Results

### Quantitative

#### Participant characteristics and migration intentions

Of the 893 invited to participate in the Doctor Emigration Project study, 523 (response rate of 58.6%) completed a questionnaire, of whom the 423 (81%) still in Ireland formed the cohort that is analysed and reported here. The mean age of participants was 32.7 years; 84% were from Ireland, 6% from another European country, 6% from Asia and 4% from other non-European countries. Females represented 58% (*n* = 240) of the sample; and 70% of the sample had undertaken a direct entry medicine programme. Forty-seven percent of participants were married and 35% had children. There was a significantly higher proportion of males with children (42%) compared to females (30%; *p* = 0.013). Forty-three percent of participants—SHOs (39%), Registrars (41%) and Specialist Registrars (49%)—stated they were not happy with their work-life balance (Additional file 2: Fig. S1).

Twenty-two percent of participants definitely or probably did not see themselves practicing medicine in Ireland for the foreseeable future, while 22% were undecided (Table 1). Canada (27%), the UK (24%) and Australia (15%) were the most popular destinations; with no significant differences by sex (Additional file 3: Table S2). A significantly higher proportion of participants intending to practice abroad (probably or definitely leave) were not happy with their work-life balance (54%), compared with those intending to practice in Ireland (32%; *P* < 0.001). Overall, 64% of participants reported having been bullied by other staff while working as a trainee doctor in Ireland, with a non-significant difference between those probably or definitely intending to leave (73%) versus those probably or definitely intending to stay (60%). Rates of intentions to leave (22–23%) were similar across grades/levels of hospital posts (Table 1) and across the 13 main training specialties (not tabulated). Of those who indicated they were Undecided and those who were probably or definitely leaving, 38% stated they were unlikely to return (32% among the Undecided and 43% among those probably or definitely leaving). A significantly higher proportion of Registrars (47%) and consultants (46%) were unlikely to return to Ireland, compared to SHOs (35%) and Specialist registrars (28%; *P* = 0.004: Additional file 4: Fig. S2).

**Table 1:** Characteristics of doctors practicing medicine in Ireland by intention to stay or leave Ireland

		Definitely stay		Probably stay		Undecided		Probably leave		Definitely leave		Total		*P*
		*n*	%	*n*	%	*n*	%	*n*	%	*n*	%	*n*	%	
Sex														
	Female	47	20	93	39	45	19	34	14	21	9	240	100	
	Male	29	17	62	36	44	26	19	11	17	10	171	100	0.454
Age														
Mean (IQR)		32.4	(29–35)	33	(30–35)	32.4	(29–34)	32.9	(30–37)	32.3	(30–35)	32.7	(30–35)	
	0–	20	20	38	38	24	24	11	11	7	7	100	100	
	30–	33	17	67	35	43	23	27	14	21	11	191	100	0.804
	35–	24	20	51	42	21	17	15	12	10	8	121	100	
Entry route^a^														
	DEM	48	20	101	41	47	19	28	11	20	8	244	100	
	GEM	12	17	26	36	20	28	7	10	7	10	72	100	0.531
	Non-EU fees	4	12	11	33	8	24	4	12	6	18	33	100	
Employment status														
Private/other^b^		20	27	33	44	11	15	8	11	3	4	75	100	
	Public	58	17	125	36	81	23	45	13	36	10	345	100	0.051
Happy with work-life balance														
	No	20	11	56	31	54	30	28	16	22	12	180	100	
	Yes	58	24	102	42	39	16	25	10	17	7	241	100	<0.001
Relationship status														
	Married	39	20	70	36	39	20	29	15	17	9	194	100	
	Other	38	17	86	39	50	23	24	11	21	10	219	100	0.662
Children														
	No	47	18	97	36	59	22	36	14	27	10	266	100	
	Yes	30	21	58	40	28	19	17	12	11	8	144	100	0.729
Current grade^c^														
	SHO	14	21	23	34	16	24	8	12	7	10	68	100	
	Registrar	19	22	28	32	18	21	14	16	8	9	87	100	
	GP trainee	6	16	15	39	13	34	2	5	2	5	38	100	
	SpR	19	13	66	44	32	21	20	13	13	9	150	100	0.191
	GP	9	18	21	41	12	24	5	10	4	8	51	100	
	Consultant	10	42	6	25	2	8	2	8	4	17	24	100	
	Other	1	33	0	0	0	0	1	33	1	33	3	100	
Bullied in Ireland														
	No	34	22	60	39	33	22	15	10	10	7	152	100	
	Yes	44	16	99	37	60	22	38	14	29	11	270	100	0.253
Return to Ireland														
	Likely			86	84	60	68	32	60	20	51	198	70	
	Unlikely			17	17	28	32	21	40	19	49	85	30	< 0.001

^a^ *DEM* medical school entrant direct from secondary school final exam, *GEM* medical school entrant with a graduate degree

^b^Private/other: other category includes research (*n* = 13), maternity leave (*n* = 16), career break (*n* = 3), not working (*n* = 1) and other (*n* = 12)

^c^Grade in order of seniority: *SHO* Senior House Officer, *GP trainee* General Practitioner trainee, *SpR* Specialist registrar, *GP* General Practitioner

### Factors influencing decision to leave

Only those who responded that they were leaving (*n* = 91), undecided (*n* = 89) or probably staying (*n* = 155) were asked about factors influencing their decision to practice medicine abroad, i.e. the 78 participants who were definitely staying were not asked these questions. We coded intentionality to practice medicine abroad into two categories: Undecided (Undecided or probably staying) and Leaving (definitely or probably) (Fig. 1). High percentages of participants in both intention categories agreed with most statements of factors influencing their decision to practice medicine abroad, as follows:

1. Working abroad was seen as more attractive than working in Ireland, because of: “better conditions abroad” (95% agreement); “better work life balance abroad” (91%); “better training opportunities abroad” (85%); “earn more abroad (76%)”; and “interview panels value training abroad” (71%); and
2. Working in Ireland was unattractive in itself, because of: “uncertainty about securing consultant posts at the end of training” (86%); “workplace understaffing” (82%); “being expected to carry out too many non-core tasks” (81%); and “consultant posts not attractive” (81%).

None of the top 16 Likert scale statements, which were re-categorised as binary variables (Agree or Disagree), discriminated significantly between intent to leave, or being undecided or probably intending to stay in Ireland. However, three of the four statements that the participants scored the lowest as reasons for leaving, two training related and one a personal reason, did discriminate significantly between those intending to leave and those undecided or probably staying: 40% of those who were undecided about practicing medicine in Ireland agreed that “the training here is of poor quality” compared to 54% of those who intended to leave (*P* = 0.021; Fig. 1). Similarly, 46% of those undecided about leaving agreed that “the supervision of training here is inadequate” compared to 60% of those intending to leave (*p* = 0.026). A significantly higher proportion of those who intended to leave (40%; *P* = 0.029) agreed with the statement “I have family or personal reasons for leaving” compared to 27% of those who were undecided (Fig. 1). Significantly higher proportions of older doctors agreed they would leave for family or personal reasons (46% of those 35 and over, compared to 31% of those aged 30–34 and 18% of those aged 29 or less; *P* = 0.002: Additional file 5: Table S3). Significantly higher proportions of married doctors agreed they would leave for family or personal reasons (42% married vs. 23% not married; *P* = 0.001). Significantly higher proportions of doctors with children (43%) agreed they would leave for family or personal reasons compared to 26% without children (*P* = 0.005: Additional file 5: Table S3).

In the primary univariable analysis that tested factors predicting trainee doctors intention to practice medicine abroad (Table 2), the only significant predictor for the binary outcome “Stayers” and “Leavers” was work-life balance. Those who were not happy with their current work-life balance were 2.51 times (OR 2.51; 95% CI, 1.53–4.10: LRT *P* < 0.001) more likely to leave (Table 2). This remained the case in the primary multivariable logistic regression when adjusting for all factors (presented in Table 1).

**Table 2:** Univariable and multivariable odds ratios of factors predicting doctors (Stayers vs Leavers) intentions to practice medicine abroad with 95% confidence intervals

	Univariable			Multivariable		
	OR	95% CI	LRT	OR	95% CI	LRT
Happy with current work-life balance						
Yes	1.00	–		1.00	–	
No	2.51	1.53–4.10	< 0.001	2.51	1.53–4.10	< 0.001

The secondary univariable analysis (Undecideds vs Leavers) considered the additional Likert scale statements as factors influencing trainee doctors’ decision to practice medicine abroad, statements which those who were definitely staying were not asked to score. Those who agreed with the statement “The quality of training available to me here is poor” were 1.82 (95%, CI 1.09–3.02: LRT *P* = 0.021) times more likely to intend to leave (Table 3). Agreement with the statement “The supervision of training here is inadequate” was also a predictor of intention to leave (OR 1.79; 95% CI, 1.07–2.98: Likelihood Ratio Test *P* = 0.025) as was the statement “I have family/personal reasons for leaving” (OR 1.80; 95% CI, 1.06–3.06: LRT *P* = 0.031). In a secondary multivariable analysis, adjusting for all variables, including the Likert scale factors influencing decisions to practice medicine abroad, agreement with the statement “The Quality of Training available to me here is poor” independently predicted intention to leave (OR 1.82; 95% CI 1.09–3.05; LRT *P* = 0.022), as did agreement with the statement “I have family/personal reasons for leaving” (OR 1.85; 95% CI 1.08–3.17; LRT *P* = 0.027) (Table 3).

**Table 3:** Univariable and multivariable odds ratios of factors predicting doctors (Undecided vs Leavers) intentions to practice medicine abroad with 95% confidence intervals

	Univariable			Multivariable		
	OR	95% CI	LRT	OR	95% CI	LRT
Quality of training available here is poor						
Disagree	1.00	–		1.00	–	
Agree	1.82	1.09–3.02	0.021	1.82	1.09–3.05	0.022
Leave for family/ personal reasons						
Disagree	1.00	–		1.00	–	
Agree	1.80	1.06–3.06	0.031	1.85	1.08–3.17	0.027
Supervision of training inadequate						
Disagree	1.00	–		–	–	–
Agree	1.79	1.07–2.98	0.025	–	–	–

### Qualitative

The in-depth qualitative interviews with doctors who were undertaking (*n* = 46) or who had recently completed (*n* = 4) postgraduate training in Ireland explored themes related to training, work-life balance, the working environment, career progression and family and personal life. Of the 50 doctors interviewed, 39 were in Ireland and 11 were abroad. Almost all of the doctors expressed their dissatisfaction with training in Ireland, discussing several factors which made it a generally unsatisfactory, frustrating and an often stressful experience, leading many to consider leaving, to train and practice medicine abroad. Issues discussed as part of the theme of training included the low availability of consultants to provide them with training; the fact that service provision demands superseded and often crowded out training; and poor supervision and weak formal training structures.

#### Lack of consultant supervisors

An overarching issue in relation to poor quality training and supervision was the absence of consultants on the wards. Some reported a general lack of oversight and validation of their clinical decisions, being left unsupervised and lack of feedback:

Definitely there wasn’t enough supervision. You’d need more consultants just for simple stuff. You’d know that you were able to do something but you just want somebody to say that you're doing it right. (Participant 42).

There aren’t enough consultants to actually watch you doing everything that they should be watching you doing and making sure that you’re doing everything right all the time. So you don’t get supervised as much as you should do really. (Participant 45).

Lack of supervision, as well as consequences for patient management, had a direct effect on trainees’ progression as those who feel a greater need for supervision were reluctant to carry out unsupervised procedures, depriving them of the same level of experience as those who felt more able or willing to undertake new procedures.

But I think that there’s a lot of trainees then that are a bit more cautious, and they would end up with less in their log book at the end of the year because, you know, they’d be looking for more support to do an operation and because of manpower issues, ….that might mean they miss out on doing the case altogether because the consultant would say ‘okay, you stay here and do this simple thing and I’ll do the complex thing’. (Participant 28)

#### The stress associated with lack of supervision during training

Those who were at earlier stages of training described the stressful nature of working and training in an unsupervised environment with such a heavy burden of responsibility.

So I was receiving acute patients in the ED (emergency department)… a week after being an intern. I can tell you without fear of contradiction that I wasn’t ready for that. You can make the argument that nothing makes you ready for that, but I certainly hadn’t had any sort of graduated exposure to the setting or guidelines or supervised performance…. (Participant 37)

You kind of learn by pure panic… you just work it out and then you’ve done it and you know the next time. But the first time you have to do something and you don’t have that support, it’s quite scary (Participant 14)

Several participants contrasted the supervised environment in other jurisdictions to the lack of supervision and accessible consultants in Ireland.

In the UK, there’s a lot more emphasis on being shown how to do something before being let off to do it. Whereas here a lot of the time, especially in surgery, it’s kind of like either you’re happy to do it by yourself unsupervised or else you don’t get to do it because the consultant will do it just because of the time pressure. (Participant 28)

Some contrasted the benefits of supervision in the UK with training in Ireland which gave doctors a strong sense of capability, albeit while sometimes practicing on a knife-edge.

I know in the UK you're much more supervised and you progress a lot more slowly and in a much more controlled fashion. But I suspect that when you come to the end of your training you might not feel as capable as you do here because you’ve had to manage difficult situations on your own, from quite early on, which is kind of dangerous. (Participant 14)

Certainly trainees here [UK] are supervised almost all of the time and it’s only really when they're on a night shift for eight hours that they actually get the chance to run a department by themselves. I suppose we got to take responsibility a bit sooner than they would. (Participant 42)

#### Service provision overrides training

Doctors asserted that training was often side-lined in favour of service provision, which they attributed to understaffing and the demands of under-resourced departments.

When I got onto a training scheme … I expected the college to be more interested in training and I suppose overall I feel the priority most of the time is . . . service provision (Participant 1).

Often described as ad hoc and arbitrary, training also depended on the attitude of individual consultant-trainers.

It just seems like that, that there’s no clear structure to it, that it’s hit or miss, that it’s very dependent on the site and on the consultant that you’d be training with. (Participant 18)

#### Lack of structure in training

Participants felt that their postgraduate medical training lacked structure and adequate learning milestones or achievements. Participants often felt this aspect of training in Ireland incentivised doctors to complete training abroad, where training schemes were better structured.

There’s more formal and structured training available in the UK than you can get in Ireland… it’s very ad hoc here and if you’re not in the right job, working for the right person, you mightn’t get much out of it. (Participant 34)

One of the consequences of the lack of clearly structured training schemes was the lack of clear and predictable career pathways:

It is about structure and career path and creating more clear progression on the way up and creating more streamlined training. (Participant 32)

#### Family personal reasons

In contrast to the quantitative findings, which highlighted reasons for leaving, many participants in qualitative interviews discussed how family reasons—wanting to be close to family or planning to have children—might or had influenced their decision to stay in Ireland.

I’d prefer to stay, mainly for family reasons. …… And I suppose here is where home is and where family is. (Participant 15)

It’s all based on my personal life rather than anything else. So family, I suppose as they get older, and friends and, you know, a realisation that the job is just a job. (Participant 16)

However, several participants felt that the structure of higher specialist training had a disruptive effect on their lives outside of work and was a key factor in the decision among some doctors to train and practice abroad. Postgraduate medical training involves rotations through different hospitals across the country and many participants highlighted how trainees were often assigned jobs without consideration for practical issues, such as organising accommodation. Participant 40 described the need to relocate frequently as “constantly uprooting your life”. Participant 11 described the difficulty of rotations when one has a family:

Like moving around over here, when you’re married and you have a family to look after. So our allocations are designed in a way that you’re allocated in one hospital for six months, so you change from one hospital to another every now and then….You hardly settle in one hospital and then you have to move on. And that, in a way, affects your family life. (Participant 11)

The financial strain of rotating around the country was noted by several trainee doctors. Some compared specialist training in Ireland with countries such as the UK and Australia where trainees could remain in one hospital or metropolitan area for the duration of training. The impact of the rotation system was noted by some as a major disincentive for doctors with young families or those who were planning families to remain in Ireland.

If people knew that their families were protected, I think you’d get a lot people who’d be much more willing to engage in training programmes in Ireland… [Rotations are] disruptive on the family. (Participant 43)

## Discussion

National medical workforce planning requires an understanding of whether the emigration of domestically trained doctors is due to the inability of the host health system to employ its graduates; and/or because of unsatisfactory or worsening working and living conditions in the host country [32]. This study, building on our earlier work [13], demonstrates Irish-trained doctors’ high levels of dissatisfaction and poor experiences with working conditions, training and career opportunities in Ireland, in comparison with the expected benefits and opportunities of working abroad. Five of the top 10 factors, where agreement ranged from 95 to 75% of participants, compared practicing abroad favourably with practicing in Ireland, including working conditions, work-life balance, training opportunities, earnings and how training abroad is perceived by interview panels in Ireland. However, most of the factors that were cited as reasons to practice abroad were reported almost equally by those likely to stay and those intending to leave. Lack of job satisfaction among doctors in the UK, Iceland and Greece has been associated with the decision to emigrate [10, 12, 15, 33–35]. Whereas, in Ireland, for the most part, high levels of dissatisfaction with training, work and career opportunities among doctors in Irish hospitals highlighted major medical workforce systemic weaknesses, but were not predictors of intention to emigrate.

Discriminating factors predicting those who were likely to leave versus those who would stay were dissatisfaction with work-life balance (Leavers 66% vs Stayers 42%); and dissatisfaction with training—agreement that the quality of training is poor (Leavers 54% vs Undecideds 40%) and agreement that supervision of training is inadequate (Leavers 60% vs Undecideds 46%). However, only “poor quality of training” remained in the logistic regression model after adjustment (Table 3), and there were generally higher levels of agreement on the experience of “inadequate supervision”, which can be viewed as a dimension of overall training quality. In the case of the small number of those who had completed their training and were categorised as consultants (*n* = 24), who had the longest experience of training in Ireland, the specific training weakness was the lack of supervision by consultant trainers. The qualitative findings reported here, based on interviews of 50 doctors conducted in 2015, provide insights into some of the root causes and consequences of this dissatisfaction, such as the stress experienced by doctors of training in an inadequately supervised environment, especially in their early years after graduation. Trainees were unable at times to get the support and supervision they needed to make critical clinical decisions and were faced with risking errors through inexperience, or not using opportunities (albeit unsupervised) to gain clinical experience.

The relationship between medical education and quality of care is complex and dynamic, and can potentially be influenced by fatigue, supervision or clinical experience [36]. A study on junior doctors-prescribing mistakes in the UK highlighted a multifaceted problem, including mistakes due to lack of knowledge and training, lack of support, time pressures, working alone without immediate access to support, the wish to appear confident, hierarchical structures and senior doctors being unapproachable or unhelpful [37]. Inadequate supervision of postgraduate trainee doctors in Ireland is a result of increasing workloads and staff shortages, exacerbated by a decade long recession and cuts to the salaries of new consultants, which has impacted on recruitment [38, 39]. Given the UK experience [36], it is also a cause of concern regarding clinical governance and patient safety.

We previously reported, in a qualitative study, dissatisfaction with working conditions in Ireland as an emigration driver [23], and a series of national reports, studies and recommendations in the last 12–15 years have sought to overhaul and improve working conditions, training structures and career opportunities [7, 25, 26, 40–43], notably the 2014 Strategic Review of Medical Training and Career Structures [42], implementation of whose recommendations is being monitored in 2015–2017 [26]. The 2014 strategic review recommended greater predictability at the outset of training in relation to locations of rotations for trainees and their families and more flexible and differentiated approaches and options during training taking account of family, and other constraints [42]. Our study provides personal insights into the experiences of postgraduate medical trainees in 2015. Poor work-life balance and the negative impact of training structures on doctors’ personal and family lives are not unique to Ireland [44–46], but are longstanding problems [47]. In the context of the global opportunities for doctors, an “inability to meet the costs of employing these professionals; or unsatisfactory or worsening working and living conditions” will drive emigration [32].

The 2014 Strategic Review of medical careers emphasised the need for recommendations to result in tangible improvements for trainee doctors’ day-to-day working lives, noting an “imbalance between training needs and service requirements” [42], which our participants helped to illustrate. High levels of burnout and dissatisfaction with work-life balance among physicians have been reported in the US and can have negative repercussions on physicians health and the quality of care they provide, issues which could be dealt with through structured mentoring programmes [45]. Trainee doctors in the UK reported how poor work-life balance detracted from learning, career progression, personal life and well-being [46, 48]. In Greece, a country similarly affected by the economic recession, low job satisfaction, fears of unemployment and a lack of standardised training [10, 34] have been reported to influence the exit of doctors from the system. Austria, with one of the highest numbers of medical graduates among OECD countries, sees 30% of its graduates emigrating [14], reasons for which include low ratings of undergraduate clinical training, unstructured postgraduate training curricula, low basic salaries, large amounts of administrative tasks and long working hours [14, 49].

In Ireland, recommendations have been made and changes are underway to restructure training, shorten training durations, and provide more predictable rotations and family-friendly training practices [26] but these changes will take time to filter through to trainee doctors’ actual experiences. In late 2016, the working group tasked with monitoring implementation reported that trainees have observed “little tangible change or impact on their day-to-day working lives and training experience” [26]. Almost two thirds (64%) of participants in our study reported having being bullied during their career, which is consistent with reports in Medical Council annual surveys of bullying in the current post of 34 and 35%, in 2014 and 2015 respectively [27, 50]. The nature of the workplace practices and experiences that participants considered as bullying were reported by us in a recent paper [51] and correspond with Lyons et al.’s definition [52]. Bullying was not a predictor of emigration intention in our study, but is a serious systemic weakness that medical workforce planners are addressing through an anti-bullying policy [51].

Understaffing and high numbers of unfilled consultant posts undermine the quality of training, especially supervised training of postgraduate doctors [26], as the qualitative findings illustrate, contributing to doctor emigration. It is notable that 47% of those who intend to leave and 53% of those who are undecided about leaving reported that, if they left, they would be unlikely to return to Ireland. Where working abroad might once have been a training step followed by a return to one’s home country, i.e. circular migration, recent research shows that the longer health professionals remain abroad the less likely they are to return [16, 17]; and therefore historical circular migration, where graduates left for training with a view to returning home, can no longer be depended upon by workforce planners. This is particularly relevant given the finding that doctors who were married, had children or were older indicated they would leave for family or personal reasons, a group far more likely to establish roots abroad due to their need to establish their careers due to their relationship commitments. These doctors have perhaps been hardest pressed throughout the economic recession, are likely to exit medical practice in Ireland in order to provide better opportunities and lifestyles for their families. Other Anglophone countries offer doctors “a 38 h working week, guaranteed protected teaching time, 16 h per day of ED [emergency department] consultant supervision, adequate remuneration for hours worked and educational activities” [53].

Our study has some limitations. Firstly, questions that asked about factors that might influence trainee doctor’s intention to emigrate were not asked of those who definitely planned to stay. If the questions had asked all trainees about their views of working, training and seeking a career in the Irish health system, rather than asking them if these were reasons for leaving to practice abroad, they might have discriminated better and would have included the full cohort of participants. Secondly, the high levels of dissatisfaction may reflect a response bias, if those who were more dissatisfied with working and training in the Irish health system were more likely to complete a questionnaire—53% of national trainees completed the Medical Council of Ireland’s “Your Training Counts” 2014 survey, of which 55% responded to our survey in 2016. It is not possible to conclude if selection bias relating to the types of trainees who responded to the Medical Council survey resulted in a sample in the doctor emigration study that was more or less likely to report an intention to practice abroad. However, there was a consistency between the qualitative and quantitative findings, as well as published studies [13, 18, 42, 43, 53] that point to the chronic systemic problems of postgraduate training structures in Ireland, which are root causes of emigration and impact on the working lives of the doctors who stay.

### ﻿Conclusion

In a globalised world, where medical graduates have a highly portable qualification, countries such as Ireland need to achieve better working and training conditions, if they are to retain their medical graduates. The under-production of doctors, or as in the case of Ireland, systemic medical workforce weaknesses—in training, work-life balance and career opportunities—provide some of the drivers to the broader phenomenon of global medical migration.﻿

## Additional files

Additional file 1:﻿ Supplementary **Table ﻿S1**: Likert scale statements. (DOCX 14 kb)

Additional file 2: ﻿Supplementary **Table S2**: Participant characteristics by gender with *P* values﻿. (DOCX 42 kb)

Additional file 3: Supplementary Figure S1: Percentage of respondents who are not happy with their work-life balance, by grade with *P* value. (XLSX 21 kb)

Additional file 4: Supplementary **Figure S2**: Percentage of respondents unlikely to return, by grade with *P* value. (DOCX 30 kb)

Additional file 5: Supplementary **Table S3**: Percentage of respondents in agreement with the statement “I would leave for family/ personal reasons”, by age, relationship status and dependents, with associated *P* values. (DOCX 15 kb)

## Abbreviations

CI: Confidence interval
GEM: Graduate Entry Medicine
LMIC: Low- and middle-income counties
LRT: Likelihood ratio test
OR: Odds ratio
WHO: World Health Organization
YTC: Your training counts
