# Supplementary material for: Factors influencing trainee doctor emigration in a high income country: a mixed methods study
Source: Hum Resour Health. 2017 Sep 25;15:66. doi: 10.1186/s12960-017-0239-7 (PMC5611654; doi:10.1186/s12960-017-0239-7)
Supplement: Supplementary file 1 — Supplementary Table ﻿S1: Likert scale statements. (DOCX 14 kb) [file 12960_2017_239_MOESM1_ESM.docx]

|  | I am considering practicing medicine abroad because: |
| --- | --- |
|  |  |
| 1 | The working hours expected of me here are too long |
| 2 | I am expected to carry out too many non-core task |
| 3 | My workplace is understaffed |
| 4 | My employer does not support me in my work |
| 5 | I am not respected by senior colleagues |
| 6 | The quality of training available to me in Ireland is poor |
| 7 | I do not have flexible training options here |
| 8 | Training pathways and duration are not predictable here |
| 9 | There are limited career progression opportunities available to me here |
| 10 | I find my work environment here is often stressful |
| 11 | The level of supervision of training available here is inadequate |
| 12 | I can earn more abroad |
| 13 | There are better training opportunities available abroad |
| 14 | I feel I need to emigrate to be competitive in Ireland |
| 15 | Consultant appointment panels value training abroad more than training in Ireland |
| 16 | I feel there is too much uncertainty around securing consultant posts in Ireland at the end of training |
| 17 | Consultant posts in Ireland are not attractive jobs |
| 18 | There are better working conditions available abroad |
| 19 | I can achieve a better work-life balance abroad |
| 20 | I have family/personal reasons for leaving |
|  |  |
