# Supplementary material for: Factors influencing trainee doctor emigration in a high income country: a mixed methods study
Source: Hum Resour Health. 2017 Sep 25;15:66. doi: 10.1186/s12960-017-0239-7 (PMC5611654; doi:10.1186/s12960-017-0239-7)
Supplement: Supplementary file 2 — ﻿Supplementary Table S2: Participant characteristics by gender with P values﻿. (DOCX 42 kb) [file 12960_2017_239_MOESM2_ESM.docx]

Supplementary figure 1: Percentage of respondents who are not happy with their work-life balance, by grade with P value
