# Supplementary material for: Factors influencing trainee doctor emigration in a high income country: a mixed methods study
Source: Hum Resour Health. 2017 Sep 25;15:66. doi: 10.1186/s12960-017-0239-7 (PMC5611654; doi:10.1186/s12960-017-0239-7)
Supplement: Supplementary file 5 — Supplementary Table S3: Percentage of respondents in agreement with the statement “I would leave for family/ personal reasons”, by age, relationship status and dependents, with associated P values. (DOCX 15 kb) [file 12960_2017_239_MOESM5_ESM.docx]

| Supplementary Table 3: Percentage of respondents in agreement with the statement "I would leave for family/ personal reasons", by age, relationship status and dependents, with associated P values | | | | | | | |
| --- | --- | --- | --- | --- | --- | --- | --- |
|  | Disagree | | Agree | | Total | | P |
|  | n | % | n | % | n | % |  |
| **Age** |  |  |  |  |  |  |  |
| 0- | 56 | 82.4 | 12 | 17.7 | 68 | 100 |  |
| 30- | 92 | 69.2 | 41 | 30.8 | 133 | 100 | 0.002 |
| 35- | 39 | 54.2 | 33 | 45.8 | 72 | 100 |  |
| Total | 187 | 68.5 | 86 | 31.5 | 273 | 100 |  |
|  |  |  |  |  |  |  |  |
| **Relationship status** | |  |  |  |  |  |  |
| Married | 71 | 57.7 | 52 | 42.3 | 123 | 100 |  |
| Other | 116 | 77.3 | 34 | 22.7 | 150 | 100 | 0.001 |
| Total | 187 | 68.5 | 86 | 31.5 | 273 | 100 |  |
|  |  |  |  |  |  |  |  |
| **Dependents** | |  |  |  |  |  |  |
| No | 136 | 73.9 | 48 | 26.1 | 184 | 100 |  |
| Yes | 49 | 57.0 | 37 | 43.0 | 86 | 100 | 0.005 |
| Total | 185 | 68.5 | 85 | 31.5 | 270 | 100 |  |
|  |  |  |  |  |  |  |  |
